# PTH [1–34] induced differentiation and mineralization of mandibular condylar cartilage

**DOI:** 10.1038/s41598-017-03428-y

**Published:** 2017-06-12

**Authors:** Mara Heather O’ Brien, Eliane Hermes Dutra, Alexandro Lima, Ravindra Nanda, Sumit Yadav

**Affiliations:** Division of Orthodontics, University of Connecticut Health Center, 263 Farmington Ave, Farmington, CT 06030 USA

## Abstract

Intermittent Parathyroid Hormone (I-PTH) is the only FDA approved anabolic drug therapy available for the treatment of osteoporosis in males and postmenopausal females. The effects of I-PTH on the chondrogenic lineage of the mandibular condylar cartilage (MCC) are not well understood. To investigate the role of I-PTH on the MCC and subchondral bone, we carried out our studies using 4 to 5 week old triple transgenic mice (Col1a1XCol2a1XCol10a1). The experimental group was injected with PTH (80 μg/kg) daily for 2 weeks, while control group was injected with saline. Our histology showed that the I-PTH treatment led to an increased number of cells expressing Col1a1, Col2a1 and Col10a1. Additionally, there was an increase in cellular proliferation, increased proteoglycan distribution, increased cartilage thickness, increased TRAP activity, and mineralization. Immunohistochemical staining showed increased expression of pSMAD158 and VEGF in the MCC and subchondral bone. Furthermore our microCT data showed that I-PTH treatment led to an increased bone volume fraction, tissue density and trabecular thickness, with a decrease in trabecular spacing. Morphometric measurements showed increased mandibular length and condyle head length following I-PTH treatment. In conclusion, our study suggests that I-PTH plays a critical role in cellular proliferation, proteoglycan distribution, and mineralization of the MCC.

## Introduction

The mandibular condylar cartilage (MCC) is a secondary cartilage, which develops from the periosteum and has 4 distinct zones: the fibrous layer, the polymorphic cell layer, the prehypertrophic cell zone, and the hypertrophic cell layer^1–4^. Beneath the hypertrophic cell layer is the subchondral bone, which closely interacts with the condylar cartilage in maintaining the physiological functions of the temporomandibular joint^3^. A physiological relationship (biological and mechanical) occurs between the subchondral bone and the MCC. Pathological alterations in the subchondral bone may be a predisposition for mineralization of the MCC and may lead to degeneration.

Parathyroid hormone (PTH), acting through the PTH receptor (PTHR1), is expressed in the MCC, in bone, and in kidney, and plays a vital role in calcium and phosphate homeostasis. PTH signaling contributes to bone and cartilage growth and remodeling^5–9^. PTH has differential effects on bone depending on the mode of administration. Continuous exposure to PTH leads to catabolic bone resorption, whereas intermittent PTH (I-PTH) leads to anabolic effects in the bone^7–9^. The chondrocytes of the MCC are mesenchymal in origin and share a lineage with osteoblasts, suggesting that I-PTH administration might stimulate cartilage synthesis.

I-PTH administration has been a commonly used drug therapy in the maintenance and healing of bone^10^, but the literature lacks the specific effects of I-PTH on the MCC. In this research, we have utilized a combination of strategies (micro-CT, histology, morphometric measurements and flow cytometry based cell sorting) to study the outcome of I-PTH treatment on the MCC. The objectives of our study were: 1) to evaluate the microscopic changes in the MCC and the subchondral bone associated with the administration of I-PTH; 2) to study the tissue level changes in the subchondral bone and calcified cartilage.

## Results

### I-PTH leads to increased expression of Col1a1, Col2a1 and Col10a1 in cell culture

**Figure 1:**
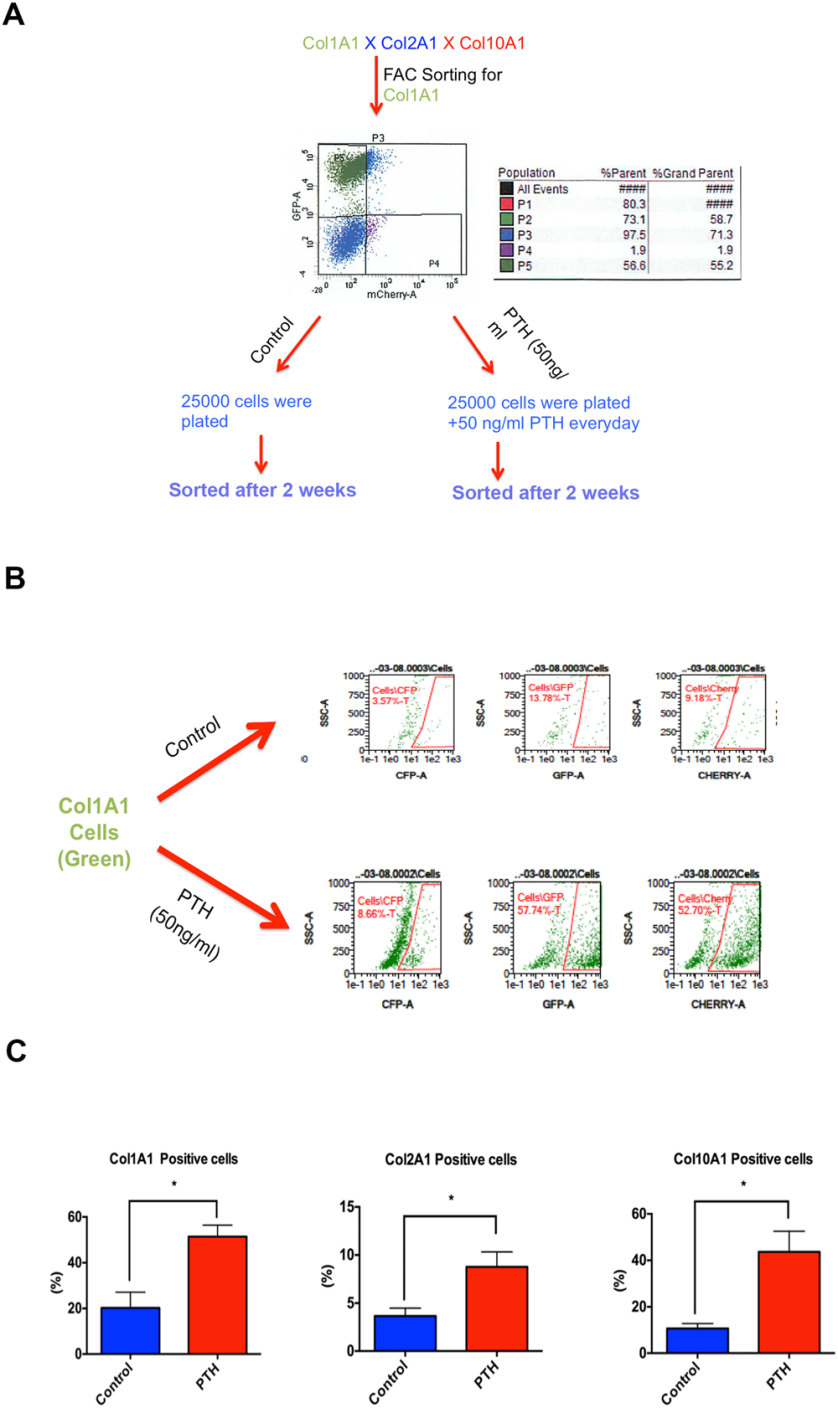
*In vitro* differentiation potential of Col1a1 expressing cells in the MCC and subchondral bone in the presence of PTH. (**A**) Col1a1 expressing cells were FACS sorted from 12 triple transgenic mice (Col1a1 X Col2a1 X Col10a1). 56.6% of the cells were Col1a1 expressing cells and equal numbers of Col1a1 cells were plated in the control and experimental groups. (**B**) Flow activated cell sorting of the Col1a1 expressing cells in the control group and experimental group (50ng/ml). (**C**) Bar graphs showing the percentage of Col1a1, Col2a1 and Col10a1 expressing cells in the control and I-PTH [1–34] groups. There was significantly increased (p < 0.05) Col1a1 expression (n = 12; 3 biological replicates and each biological replicate had 4 mice); CI for control group: 3.19–37.35; CI for PTH group: 38.98–63.84), Col2a1 expression (n = 12; 3 biological replicates and each biological replicate had 4 mice); CI for control group: 1.64–5.69; CI for PTH group: 4.90–12.63) and Col10a1 expression (n = 12; 3 biological replicates and each biological replicate had 4 mice); CI for control group: 5.34–15.89; CI for PTH group: 21.61–65.71) in cells after daily treatment with 50ng/ml of PTH [1–34] in the experimental group.*Statistically *s*ignificant difference between control and PTH [1–34] groups.

Cells from the MCC of triple collagen transgenic mice (Col1a1 X Col2a1 x Col10a1) were freshly isolated and PTH [1–34] (50ng/ml) was added daily for 2 weeks. We then examined the effects of PTH [1–34] on the expression of Col1a1-green cells, Col2a1-blue cells and Col10a1-red cells in comparison to control (Fig. 1A and B). The presence of PTH [1–34] leads to significantly increased expression of Col1a1 positive cells (60.5%; p < 0.05), Col2a1 positive cells (58.15%; p < 0.05) and Col10a1 positive cells (75.69%; p < 0.05) (Fig. 1B and C), indicating increased proliferation and differentiation (Fig. 1C) *in vitro*.

### I-PTH leads to increased bone volume fraction, tissue density and trabecular thickness

**Figure 2:**
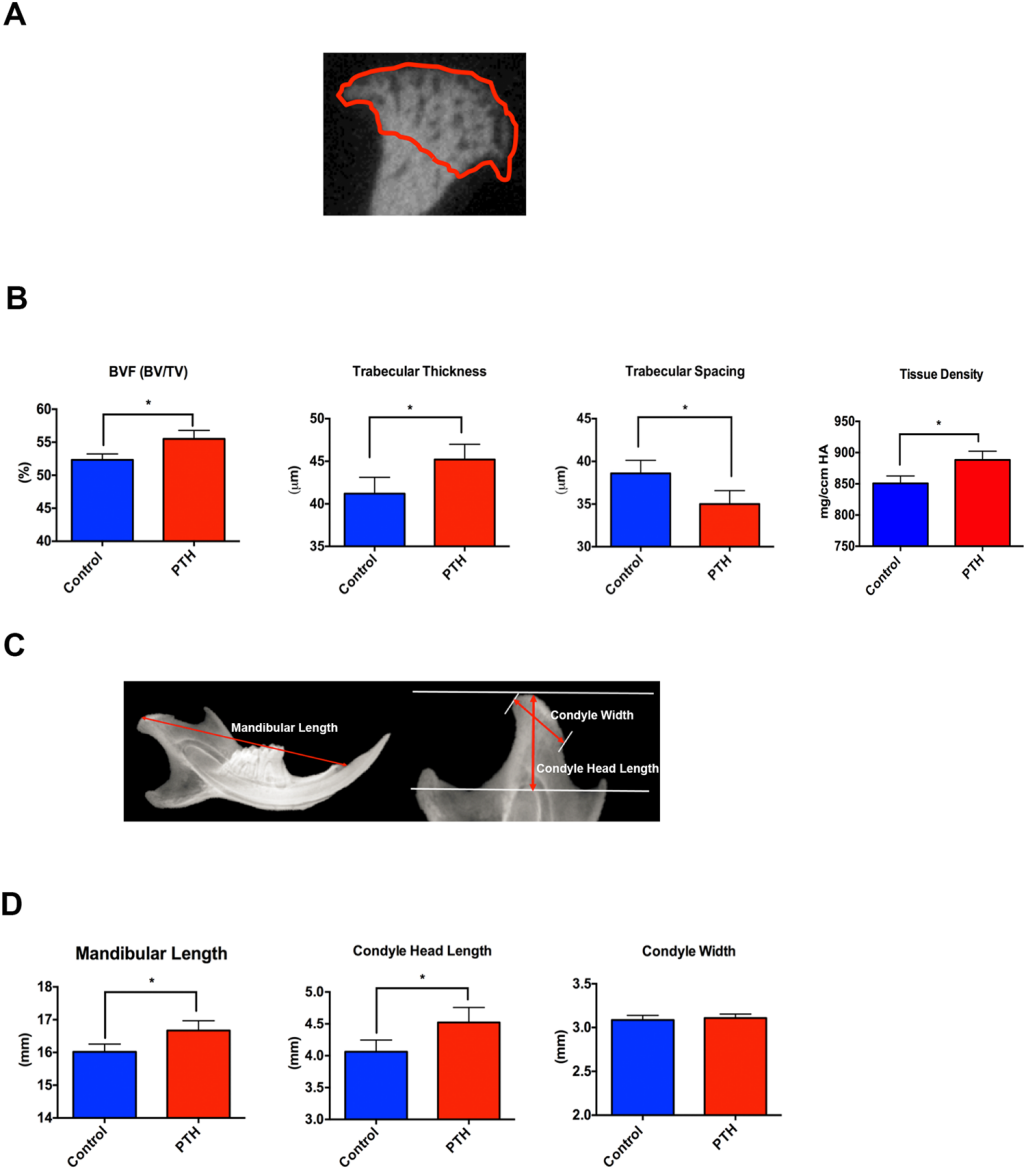
Increased bone volume, trabecular thickness and mandibular length in the I-PTH injected mice. (**A**) Region of interest for measurement of bone volume fraction, tissue density, trabecular thickness and trabecular spacing. (**B**) Histograms showing the significant increase (p < 0.05) in bone volume fraction (BVF; n = 6; CI for control group: 51.9–53.45; CI for I-PTH group: 53.89–57.11) trabecular thickness (n = 6; CI for control group: 42.98–45.82; CI for I-PTH group: 44.98–47.82) and tissue density (n = 6; CI for control group: 836.2–865.4; CI for I-PTH group: 870.7–905.7) and significant decrease (p < 0.05) in trabecular spacing (n = 6; CI for control group: 31.76–34.24; CI for I-PTH group: 35.18–38.02) in the experimental group (I-PTH). (**C**) Morphometric measurements performed with Faxitron x-ray images of control and experimental (I-PTH) groups. (**D**) Histograms showing a significant increase (p < 0.05) in the mandibular length (n = 6; CI for control group: 15.73–16.31; CI for I-PTH group: 16.29–17.04) and condyle head length (p < 0.007) (n = 6; CI for control group: 3.83–4.29; CI for I-PTH group: 4.23–4.81) in the experimental group (I-PTH). *Statistically *s*ignificant difference between control and I-PTH groups.

We used micro-computed tomography (micro-CT) to assess the quantitative changes in the calcified cartilage and subchondral bone of mice that received I-PTH or saline solution injections. Bone volume, tissue density, trabecular thickness and trabecular spacing were evaluated (Fig. 2A). Two weeks after the administration of I-PTH, the mice in the experimental group showed a significant increase in the bone volume fraction (5.7%; p < 0.04) (Fig. 2B), tissue density (10.41%; p < 0.005) (Fig. 2B), trabecular thickness (6.03%; p < 0.05) (Fig. 2B) and a significant decrease in trabecular spacing (9.84%; p < 0.05) (Fig. 2B) when compared to the control mice.

### I-PTH leads to increased mandibular length and condylar head length

Morphometric measurements were performed in radiographic images of mandibles from the experimental and control groups. There was a significant increase in the mandibular length (2.67%; p < 0.05) (Fig. 2C and D) and in the condyle head length (11.32%; p < 0.007) in the I-PTH injected group (Fig. 2C and D); however the condylar width was not significantly different between control and I-PTH groups (p = 0.66) (Fig. 2C and D).

### I-PTH leads to increased proliferation and differentiation in the mandibular condylar cartilage

**Figure 3:**
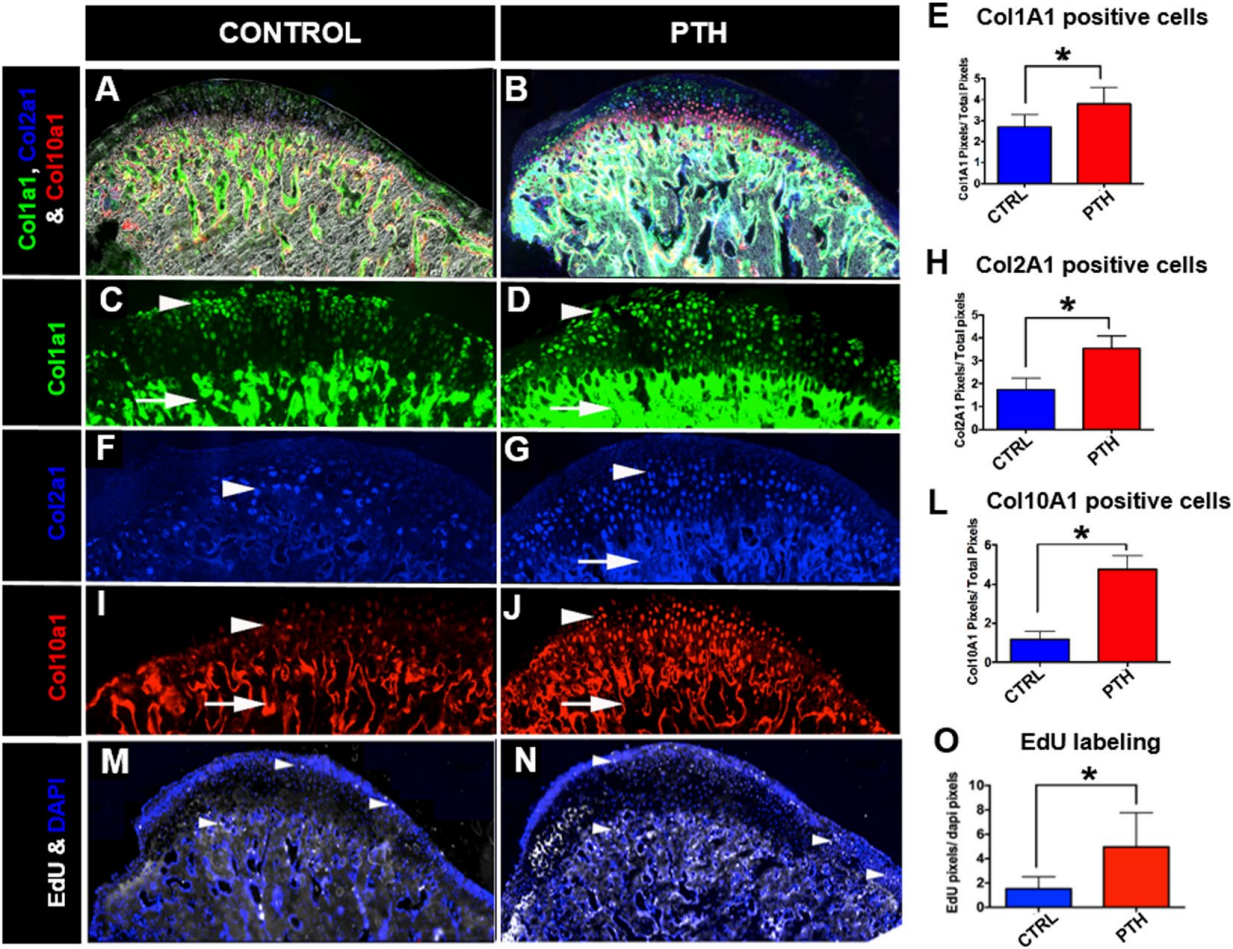
I-PTH leads to increased Col1a1, Col2a1 and Col10a1 expressing cells. Sagittal sections of the MCC and subchondral bone of triple transgenic mice (Col1a1 X Col2a1 X Col10a1) in the control (**A**) and I-PTH (**B**) groups. Col1a1 cells in the MCC (arrowhead) and subchondral bone (arrow) in the control (**C**) and I-PTH (**D**) groups. Col2a1 cells in the prehypertrophic zone (arrowhead) of the MCC in control (**F**) and I-PTH (**G**) groups. Presence of Col2a1 cells in subchondral bone of experimental mice (arrow). Col10a1 cells in the hypertrophic zone (arrowhead) of the MCC in control (**I**) and I-PTH (**J**) groups. The red color (arrow) in the subchondral bone is alizarin complexone bone label (I–J). Histograms showing the significant increase in Col1a1 (p < 0.0004) (**E**), Col2a1 (p < 0.000) (H) and Col10a1 (p < 0.000) (**L**) positive pixels in the I-PTH group relative to controls. Sagittal sections stained for EdU in the control (**M**) and I-PTH (**N**) groups. EdU positive cells in the MCC (arrowhead) and subchondral bone (arrow). (**O**) Histogram showing the significant increase (p < 0.000) in EdU positive pixels (n = 5; 3 histological sections from each mice; CI for control group: 0.81–2.22; CI for I-PTH group: 2.96–6.96) in the experimental group (I-PTH). *Statistically *s*ignificant difference between control and I-PTH groups.

Our histology showed that fluorescent Col1a1-green cells are present in the proliferative layer of the MCC (Fig. 3C and D), whereas fluorescent Col2a1-blue cells are present in the prehypertrophic and hypertrophic zone (Fig. 3F and G), and fluorescent Col10a1-red cells are present in the hypertrophic zone of the MCC (Fig. 3I and J). We observed an increase in the number of Col1a1 positive cells (Fig. 3E; 28.57%; p < 0.004), Col2a1 positive cells (Fig. 3H; 50.56%; p < 0.000) and Col10a1 positive cells (Fig. 3L; 75.73%; p < 0.000) in the MCC of mice that received I-PTH in comparison to the control mice. Our cell proliferation assay has detected EdU positive cells primarily in the proliferative zone of the MCC (Fig. 3M and N), in both I-PTH and control groups. However, we found a significant increase in the number of EdU positive cells in the MCC of the experimental group (Fig. 3O; 69.41%, p < 0.000), indicating enhanced cell proliferation after I-PTH administration.

### I-PTH leads to increased mineralization in the MCC and increased bone turnover in the subchondral bone

**Figure 4:**
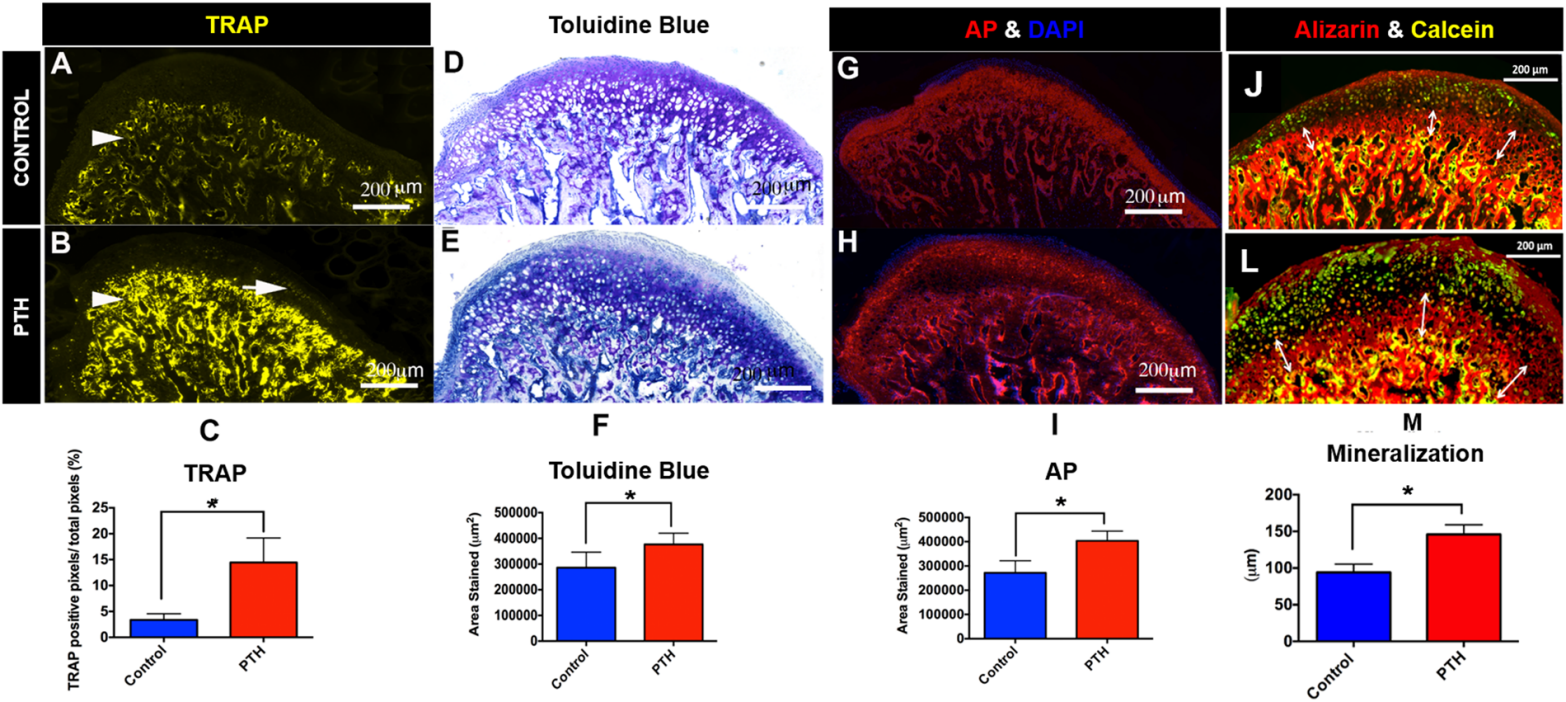
Increased osteoclastic activity and proteoglycan secretion with I-PTH injection. (**A**) Sagittal section stained for TRAP activity in the control group. TRAP stained cells are chiefly present in the subchondral bone (arrow head). (**B**) Sagittal section stained for TRAP activity in the experimental group (I-PTH). TRAP stained cells are present both in the subchondral bone (arrow head) and in the MCC (arrow). (**C**) Quantification of TRAP positive pixels over the subchondral bone area shows significantly increased (p < 0.05) TRAP positive pixels in the experimental group (I-PTH) (n = 5; 3 histological sections from each mouse; CI for control group: 2.63–4.08; CI for I-PTH group: 11.60–17.30). Sagittal sections stained for toluidine blue in the control (**D**) and I-PTH (**E** groups. **F**) Quantification shows increased (p < 0.05) toluidine blue stained area in the experimental group (I-PTH) (n = 5; 3 histological sections from each mouse; CI for control group: 251107–320973; CI for I-PTH group: 350897–401743). Sagittal sections stained for alkaline phosphatase activity in the control (**G**) and I-PTH (**H**) groups. (**I**) Quantification shows increased (p < 0.05) alkaline phosphatase stained area in the experimental group (I-PTH) (n = 5; 3 histological sections from each mouse; CI for control group: 243026–300483; CI for PTH group: 380180–426889). Alizarin complexone and calcein labeling in sagittal sections of control (**J**) and I-PTH (**L**) groups. Histogram represents distance between calcein and alizarin complexone labels. Increased (p < 0.05) labeling was seen in the I-PTH group. *Statistically *s*ignificant difference between the control and I-PTH groups.

**Figure 5:**
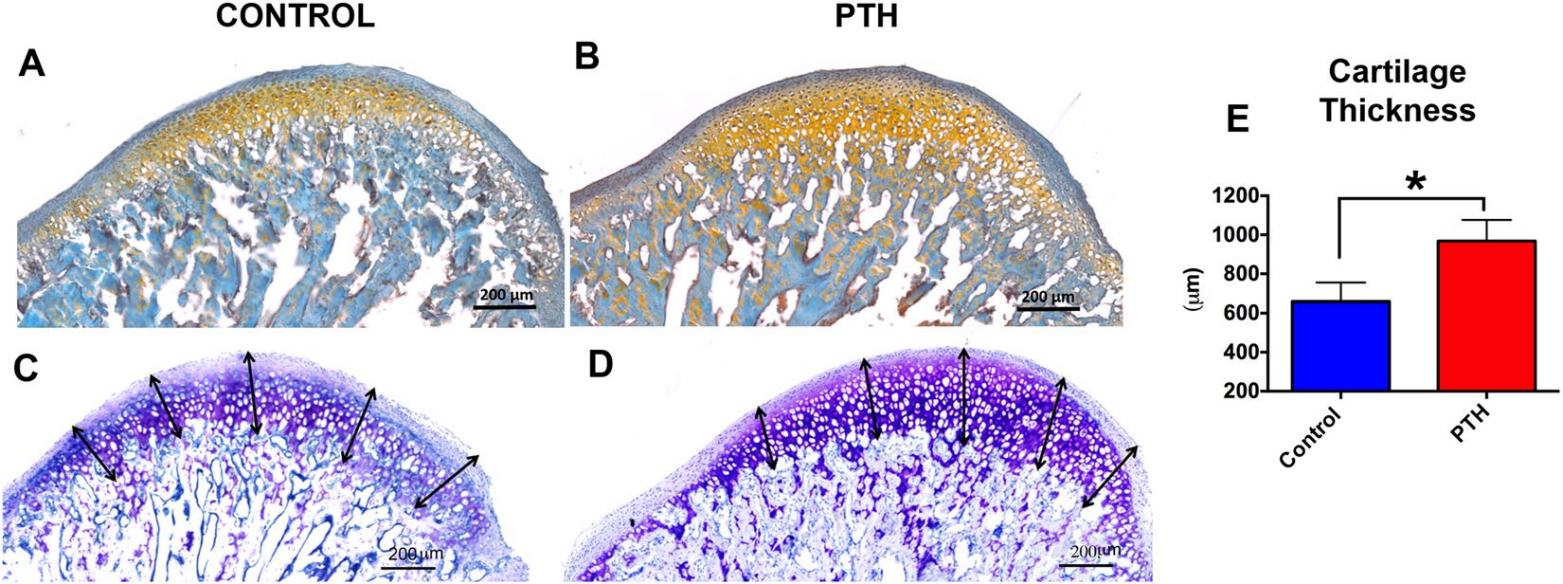
Increased Safranin O and cartilage thickness in the I-PTH group. Sagittal sections of condyles of control (**A**) and I-PTH (**B**) stained for Safranin O. Toluidine blue distance map in control (**C**) and I-PTH (**D**) groups. Histogram showing increased (p < 0.0001) thickness of cartilage in the I-PTH group in comparison to control. *Statistically *s*ignificant difference between the control and I-PTH groups.

We studied bone turnover in the MCC and the subchondral bone of experimental and control mice by TRAP staining. We observed TRAP positive cells primarily located in the subchondral bone in the control group (Fig. 4A, arrowhead), whereas TRAP positive cells were present both in the subchondral bone and the MCC in the I-PTH group (Fig. 4B, arrowhead and arrow). TRAP activity in the MCC of I-PTH injected mice was primarily present in the proliferative and prehypertrophic zones of the MCC. Our quantification showed a significant increase in the percentage of TRAP positive pixels (p < 0.0001) in the I-PTH injected group when compared to the control group (Fig. 4C). Next, we analyzed proteoglycan distribution by Toluidine Blue and Safranin O staining, which revealed increased proteoglycan secretion in the MCC of I-PTH injected group in comparison to control, as illustrated by a significant increase in the stained area (p < 0.0004) (Fig. 4D,E and F; Fig. 5A and B). In addition, we measured the thickness of the cartilage in the Toluidine Blue sections and found significantly greater thickness (p < 0.0001) in the I-PTH group in comparison to control (Fig. 5C,D and E).

Similarly, quantification of alkaline phosphatase (an enzymatic indicator of mineralization) showed significantly increased stained area (p < 0.0001) in the I-PTH injected animals (Fig. 4G,H and I) relative to control, suggesting increased mineralization of the MCC and subchondral bone after I-PTH treatment. Additionally, we found that there was a significant increase in the mineralization of the subchondral bone, as determined by alizarin complexone labeling, in the I-PTH group (Fig. 4J,L and M) compared to the control group.

### I-PTH leads to increased expression of pSMAD158 and VEGF expression

**Figure 6:**
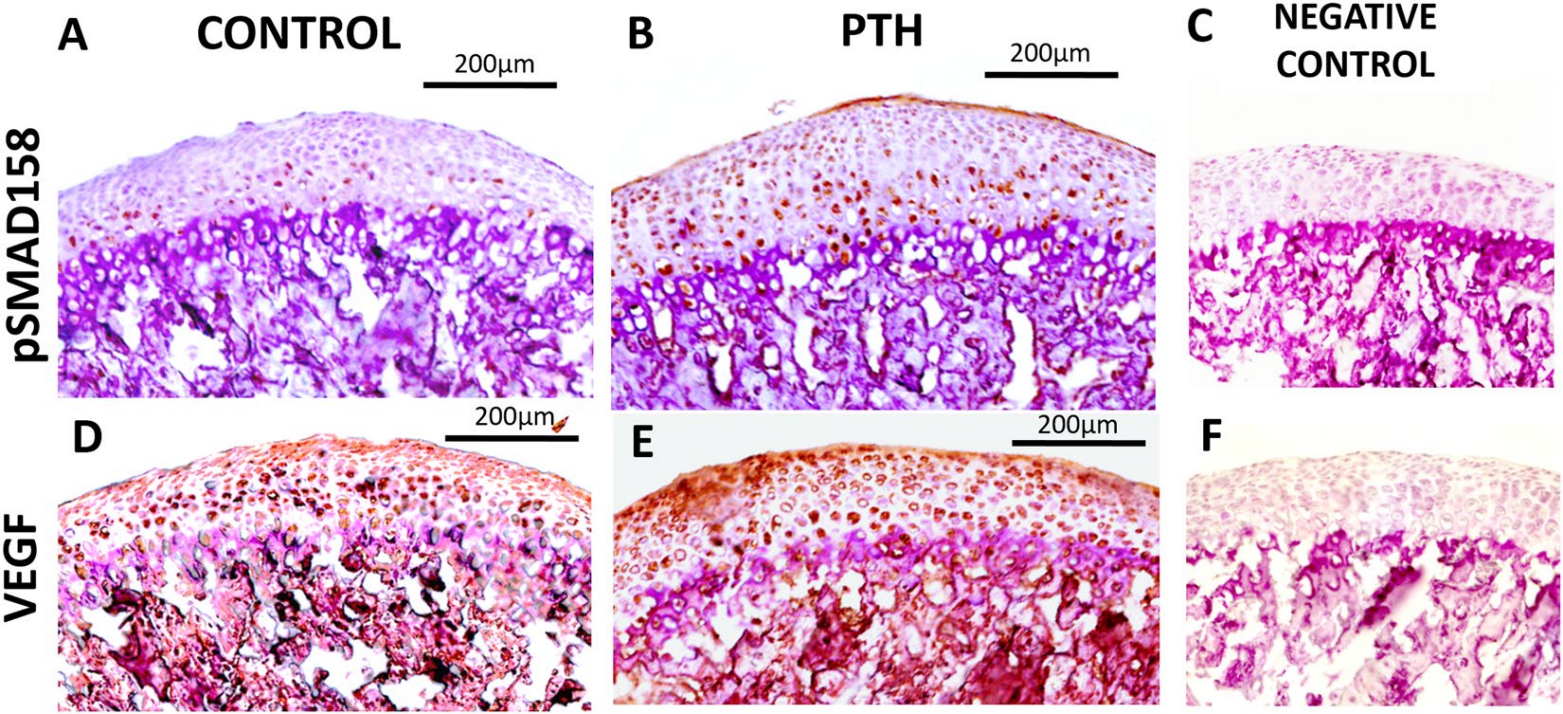
Increased expression of pSMAD158 and VEGF in the I-PTH group. Immunohistochemistry for pSMAD158 and VEGF in sagittal sections of the MCC and subchondral bone of control (**A**) and I-PTH (**B**) groups. (**C**) Negative control for pSMAD158 immunostaining. (**D**) Immunohistochemistry for VEGF in sagittal sections of control (**D**) and I-PTH (**E**) groups. (**F**) Negative control for VEGF immunostaining.

Our immunohistochemistry showed increased expression of pSMAD158 in the MCC and subchondral bone of the experimental group, relative to the control group (Fig. 6A and B). Similarly, there was also an increase in the expression of VEGF in the not only in the MCC but also in the subchondral bone of the I-PTH administered group (Fig. 6D and E).

## Discussion

To our best knowledge, this is the first study to demonstrate that systemic intermittent administration of PTH leads to an alteration of the microarchitecture of the subchondral bone and an increased mineralization/calcification of the MCC. The major findings of this study are that I-PTH increases cell proliferation and differentiation in the MCC and significantly increases turnover of the subchondral bone. Furthermore, I-PTH treatment significantly increases the zone of the calcified cartilage, thus increasing the condylar head length and total mandibular length.

PTH is primarily regarded as a regulator of calcium homeostasis but it has also been shown to enhance bone-remodeling^11–13^. PTH is currently the only anabolic drug approved for the treatment of osteoporosis^14^. Furthermore, PTH has been shown to repair the osteochondral defects of the articular cartilage^15–17^. In our research, I-PTH administration exerts an anabolic effect on subchondral bone modeling by increasing the activity and number of osteoblasts, as indicated by an increase in the number of Col1a1 cells in the I-PTH group when compared to the control. Similar effects on the anabolic modeling of the subchondral bone in the articular cartilage by PTH have been observed by other investigators^17–19^. The exact mechanism for bone anabolic responses due to PTH is not clearly understood, however it has been speculated that PTH leads to an increase in the number of mature osteoblasts, possibly due to a decrease in the apoptosis of mature osteoblasts^20^. Furthermore, it has been shown that PTH increases the OPG/RANKL ratio, suggesting anabolic modeling of the subchondral bone^17^.

Our results showed increased hypertrophic differentiation of chondrocytes both *in vitro* and *in vivo* with PTH treatment. Moreover, our histology showed increased calcification/mineralization of the cartilage. Increased cartilage mineralization and hypertrophic differentiation are predisposing factors leading to osteoarthritis of the cartilage^21^. The molecular mechanism regulating the progression of osteoarthritis is unclear, however chondrocyte differentiation is regarded as an established factor in the progression of osteoarthritis^22^. Further, we studied bone turnover and mineralization of the MCC and the subchondral bone. TRAP activity was primarily detected in the subchondral bone and mineralized cartilage and was significantly increased with I-PTH treatment. TRAP enzymatic activity is directly correlated with osteoclast/chondroclast activity as TRAP enzyme is released by multinucleated osteoclasts and activated by Cathepsin K^23^. In order to understand the increased bone turnover indicated by an increase in TRAP activity at the subchondral bone after I-PTH treatment, we performed immunohistochemistry for VEGF, an angiogenic stimulator known to attract osteoclasts/chondroclasts during endochondral ossification^24^. We found increased VEGF expression, consistent to an increase in TRAP activity. It has been showed that VEGF is expressed by the hypertrophic cells of the MCC and regulates the formation and invasion of new blood vessels, which in turn leads to the advancement of the mineralization front^25, 26^. In our study, there were more VEGF positive cells with the administration of I-PTH and, probably because of the increased angiogenesis, there was more cartilage mineralization in the MCC of the I-PTH treated animals. Additionally, we used alkaline phosphatase as an enzymatic indicator of mineralization and found significantly higher levels of mineralization in the MCC within the I-PTH treated group, when compared to the control group. We further noticed increased proteoglycan secretion in the MCC of the I-PTH group, which was probably the result of an increase in the number of mature chondrocytes (Col2a1 cells), as these cells are responsible for the secretion of proteoglycans. It has been shown that the MCC and the underlying subchondral bone do communicate over the calcified tissue barrier through molecular cross talk^27, 28^. However, it is not clear whether the changes in the subchondral bone occur as a cause or consequence of cartilage degeneration.

It has been clearly demonstrated that I-PTH exerts its anabolic effects on mineralized tissue through interaction with local factors including BMPs, Wnts, TGFβ and IGF-1. TGFβ signaling pathway (including pSMAD158) plays a critical role in the maintenance of cartilage homeostasis and structural integrity. TGFβ stimulates early events in chondrogenesis including cell proliferation, cell maturation and cell differentiation^29, 30^. Our immunohistochemistry data showed that I-PTH treatment induced enhancement of the pSMAD158 signaling pathway, which may have resulted in more Col1a1-positive, Col2a1-positive, and Col10a1-positive cells both in cell culture and *in vivo*. Furthermore, it has been shown that an increase in TGFβ signaling leads to an increase in cartilage proteoglycans, and our results showed that with I-PTH treatment there was an increase in proteoglycan area in the MCC.

PTH receptors have been identified both on osteoblast and endothelial cells^31, 32^. Studies have shown that PTH administration increases the expression of VEGF, thus augmenting blood flow and creating a favorable microenvironment for anabolic bone effects^33^. Furthermore, it has been shown that injecting VEGF antibody neutralizes the anabolic bone effects. Our research has shown that I-PTH administration leads to an increase in VEGF expression in the MCC and the subchondral bone, followed subsequently by an increase in bone volume fraction, an increase in trabecular number and a decrease in trabecular spacing^34^. More importantly there was an increase in Col1a1 expression (3.6 Kb promoter, expressed by osteoblasts) in the subchondral bone. Prisby *et al*. showed that the PTH dependent osteoanabolic response of bone is due to intact VEGF signaling, which in turn increases the osteoblast number and function^33^. Jilka *et al*. and Langer *et al*. showed that rodents given daily injections of PTH exhibit increased vascularity near the sites of bone formation. Taken together, these observations along with our findings, indicate the need for future studies to define the role of VEGF and the importance of new blood vessel formation in mediating the anabolic response to I-PTH treatment.

A possible limitation of this study is that we used growing mice for our experiments (the MCC is expected to grow until the age of 8–10 weeks in mice), whereby the age related growth of the MCC might augment the magnitude of the I-PTH induced bone anabolic response. Our future research will focus on administering I-PTH in adult animals, after the growth of the MCC is complete. Furthermore, we are focusing on studying the effects of I-PTH on the MCC and the subchondral bone before and after ovariectomy, as PTH is the only FDA approved anabolic drug for osteoporosis and the effects of the I-PTH on the MCC and the subchondral bone are yet not reported.

## Conclusions

In summary, the present study showed that I-PTH treatment increases the number of Col1a1-positive cells, Col2a1-positive cells and Col10a1-positive cells *in vitro* and *in vivo*. Furthermore, this treatment leads to increased bone volume fraction, tissue density and trabecular thickness of the subchondral bone. Our *in vivo* experiments also showed increased cellular proliferation and differentiation. Furthermore, we showed there is an increase in proteoglycan distribution with a concomitant increase in MCC mineralization. Increased chondrocytes differentiation and increased mineralization are predisposing factors for cartilage degeneration. However, to conclude convincingly we need to study the effects of long-term administration of I-PTH on adult animals. To our knowledge, this is the first study looking into the effects of I-PTH on the MCC and subchondral bone. These unrecognized effects of I-PTH on the MCC should incite future studies with prolonged observational periods before being translated into patient care.

## Materials and Methods

### Ethical Statement

The Institutional Animal Care Committee of the University of Connecticut Health Center approved the experimental protocol involving the transgenic mice in this study. Our study followed the ARRIVE guidelines^35^ for *in vivo* experiments using these mice.

### Transgenic Mice

We used thirty-four 5 to 6-week-old male transgenic mice (Col1a1 X Col2a1 x Col10a1) on a CD-1 background for this study. The GFP transgenes used in this study have been previously described^36, 37^. The Col1a1-GFP cyan contains a 3.6-kb fragment of the rat type 1 collagen promoter that is strongly expressed in bone and the MCC. The Col2a1-CFP contains a 1-kb fragment of the type 2-collagen promoter that is expressed in the pre-hypertrophic zone of the MCC. The Col10a1-RFP is fused to mCherry fluorescent protein and is expressed in the hypertrophic zone of the MCC^38^. The mice were bred and genotyped so that all three GFP reporters were present to make the triple transgenic mice (Col1a1, Col2a1 and Col10a1) used in this study

### *In-vitro* Methods

Twelve, 5 to 6-week-old male transgenic mice (Col1a1 X Col2a1 x Col10a1) were used in this part of our study. Immediately after euthanization, the MCC along with the subchondral bone was dissected under a dissection microscope and the MCC was collected. Cells from the MCC were harvested by digestion with collagenase D (4 mg/ml) (Roche Diagnostics, Mannheim, Germany) and dispase (4 mg/ml) (Gibco, Grand Island, NY, USA). Single cell suspensions were prepared by re-suspending the cell pellets in 2 ml of fixed staining medium (HBSS + 10 ml of HEPES + 2%FBS) and passing through an 18-gauge needle followed by filtration through a 100 µm strainer.

Single cell suspensions were sorted for Col1a1-positive cells (green cells-EGFP) on an LSRII flow cytometer (BD Biosciences, San Jose, CA, USA). Twenty-five thousand cells were plated in both experimental and control groups. The experimental group cells received 50ng/ml of PTH [1–34] every day for 2 weeks. After 2 weeks, cells were analyzed in both groups using a LSRII flow cytometer with BD FACS Diva analysis software (BD Biosciences, San Jose, CA, USA). Gates for single cells and debris exclusion were made based on light scatter properties. For each sample, a minimum of 50,000 events was collected. Green fluorescent protein was excited using a 50 mW 488 nM laser and fluorescence was detected from 505–550 nM. Cyan fluorescent protein was excited using a 100 mW 405 nM laser and fluorescence was detected from 425–475 nM. mCherry was excited using a 100 mW 561 nM laser and fluorescence was detected from 600–620 nM. The percentage of Col1a1-green, Col2a1-blue and Col10a1-red positive cells was determined in the control (saline) and experimental groups (PTH [1–34] treatment for 2 weeks).

### *In-vivo* Methods

Mice were divided into 2 groups: (1) Experimental (I-PTH) group (n = 12): PTH [1–34] (80 μg/kg body weight, Prospec, East Brunswick, NJ, USA) was injected intraperitoneally daily for two weeks; (2) Control group (n = 10): saline was injected intraperitoneally daily for two weeks. All the mice were injected with calcein (3 μg/kg body weight) on the 11^th^ day and alizarin complexone (3 μg/kg body weight) on the 13^th^ day. Further, mice were injected with EdU (5-ethnyl-2′-deoxyuridine, Life Technologies, Grand Island, NY, USA) (30 mg/kg body weight) 24 hours before euthanization. All the animals in the control and experimental groups were healthy and gained weight during the entire duration of the study. Mice were euthanized 24 hours after the last injection of PTH [1–34] or saline.

### Tissue Preparation and Histological Sectioning

The mandibles were dissected free by cutting the muscular attachment without scrapping the cartilage of the condyle. The MCC along with the subchondral bone were fixed for 24 hours in 10% formalin, placed in 30% sucrose buffered in PBS overnight and embedded in cryomedium (Thermo Shandon, Pittsburgh, PA, USA) using disposable base molds (Thermo Shandon, Pittsburgh, PA, USA). The medial surfaces of the samples were embedded against the base of the mold, parallel to the floor of the mold. Specimens were stored at −20 °C or −80 °C before they were sectioned using a Leica cryostat (Nussloch, Germany). Frozen sagittal sections of the condyles (5–7 µm) were performed and transferred to slides using a tape transfer method^39^.

### Micro-CT

The MCC and subchondral bone of condyles of I-PTH and control mice were analyzed using micro-computerized tomography (micro-CT) (SCANCO Medical AG, Brüttisellen, Switzerland). The samples (n = 6 per group) were scanned in 70% alcohol and serial tomographic projections were acquired at 55 kV and 145 µA, with a voxel size of 6 µm and 1000 projections per rotation were collected at 300,000 µs. In order to distinguish calcified tissue from non-calcified tissue, an automated algorithm using local threshold segmented the reconstructed grey scale images. Our region of interest was the mushroom shaped head of the condyle. Bone volume fraction (BVF (%)), trabecular thickness (Tb.Th (µm)), trabecular spacing (Tb.Sp (µm)) and tissue density (mg/ccm HA) were assessed.

### Morphological Measurement

Radiographs of the mandibles were taken with a MX20 Radiography System (Faxitron X-Ray LLC, Lincolnshire, IL, USA) at 26 Kv for 5 seconds. We performed morphometric measurements on x-rays of mandibles of mice subjected to I-PTH administration and controls. The parameters measured and compared were: 1) mandibular length (condylion to incisor process) 2) Condyle head length (the perpendicular distance from condylion to a line traced from the sigmoid notch to the deepest point in the concavity of the mandibular ramus) and 3) Condyle head width (distance from the most anterior to the most posterior point of the condylar articular surface). Measurements were performed using Digimizer^®^ Image software (MedCalc Software, Mariakerke, Belgium). Each parameter was measured three times and the average was calculated.

### Histomorphometry and Histological Staining

Our histological sections were stained and analyzed following a previously described protocol^40^. The 5–7μm MCC and subchondral sagittal sections remained adhered to glass slides through all of the processes of staining and imaging. The first step was to image for fluorescent signals Col1a1 (green), Col2a1 (blue) and Col10a1 (red) and the bone labels alizarin complexone (red) and calcein (green). Baseline imaging of the sections was performed with the observer ZI fluorescent microscope (Carl Zeiss, Thornwood, NY, USA) using a yellow fluorescent protein filter (eYFP, Chroma Cat 49003ET, EX: 500/20, EM: 535/30), a cyan fluorescent protein filter (CFP, Chroma Cat 49001ET, EX: 436/20, EM: 480/40), and a RFPcherry filter that was also used for detecting alizarin complexone staining (mCherry, Chroma Cat 49009ET, EX: 560/40, EM: 630/75). In the next step, the coverslip was removed by soaking slides in PBS and sections from both the control and experimental groups (I-PTH) were stained for EdU (Life Technologies, Grand Island, NY, USA) and imaged. Subsequently, sections were stained for Tartrate Resistant Acid Phosphatase (TRAP) using ELF97 (Life Tech, Waltham, MA, USA), which generates a yellow fluorescent signal. After imaging for TRAP, the coverslip was removed and the same slide was stained for alkaline phosphatase activity using a fluorescent fast red substrate (Sigma, St. Louis, MO, USA) and for cell nuclei using DAPI (Thermo Fisher Scientific, Waltham, MA, USA) and reimaged. Finally, the slide was rinsed in distilled water and stained with Toluidine Blue (TB) to examine proteoglycans, and reimaged using bright field microscopy.

Immunohistochemistry for SMAD158 (EMD Millipore, Billerica, MA, USA) and VEGF (Vascular Endothelial Growth Factor, ABCAM, Cambridge, MA, USA) were performed.

Details on all histological staining can be found in Supplementary Data.

### Histological analysis and quantification

Col1a1, Col2a1 and Col10a1 expression in sagittal sections of condyles was quantified using Adobe Photoshop (Adobe Systems Incorporated, San Jose, CA, USA) by counting the green, blue and red pixels within the MCC (Supplementary Figure 1A). A percentage of green, blue and red pixels in the MCC were obtained by dividing the number of green, blue and red pixels over the total number of pixels of the entire MCC. We examined TRAP activity in the MCC and subchondral bone by counting the number of yellow pixels and dividing by the total number of pixels in the subchondral bone region. Cell proliferation was quantified by counting EdU and DAPI positive pixels in the proliferative zone of the MCC and then calculating the percentage of EdU positive pixels over DAPI positive pixels. Alkaline phosphatase distance mapping was analyzed using Digimizer^®^ Image software and measurements were performed from the outer cellular layer of the MCC to the tidemark (in six different locations in the entire MCC). Finally, Toluidine Blue stained area were evaluated using Digimizer^®^ Image software.

### Statistical Analyses

Descriptive statistics were used to examine the distribution of bone volume fraction, trabecular thickness, trabecular spacing, tissue density, morphometric measurements and histological analysis. A one-sample Kolmogorov-Smirnov test was used to examine the normality of data distribution. Outcomes were compared between the control and the experimental PTH [1–34] group. Statistically significant differences were determined by the unpaired t-test (Student t-test), with post hoc analysis by Mann-Whitney U test. In the *in vitro* experiments, the percentage of Col1a1-green, Col2a1-blue and Col10a1-red positive cells was determined in the control and experimental (intermittent PTH [1–34]) groups. All statistical tests were two sided and a p-value of <0.05 was considered to be statistically significant. Statistical analyses were computed using Graph Pad Prism (San Diego, CA, USA).

## Electronic supplementary material

Histological Staining
