# Supplementary material for: PTH [1–34] induced differentiation and mineralization of mandibular condylar cartilage
Source: Sci Rep. 2017 Jun 12;7:3226. doi: 10.1038/s41598-017-03428-y (PMC5468307; doi:10.1038/s41598-017-03428-y)
Supplement: Supplementary file 1 — Histological Staining [file 41598_2017_3428_MOESM1_ESM.doc]

**PTH [1-34] induced differentiation and mineralization of mandibular condylar cartilage**

Mara Heather O’ Brien

Post Doctoral Researcher

Division of Orthodontics

University of Connecticut Health Center

Email id: [obrien@uchc.edu](mailto:obrien@uchc.edu)

Eliane Hermes Dutra

Assistant Professor

Division of Orthodontics

University of Connecticut Health Center

Email id: [edutra@uchc.edu](mailto:edutra@uchc.edu)

Alexandro Lima

Orthodontic Resident

Division of Orthodontics

University of Connecticut Health Center

Email Id: [allima@uchc.edu](mailto:allima@uchc.edu)

Ravindra Nanda

Professor & Head

Division of Orthodontics

University of Connecticut Health Center

Email id: [nanda@uchc.edu](mailto:nanda@uchc.edu)

*Sumit Yadav

Assistant Professor

Division of Orthodontics

University of Connecticut Health Center

Email id: [yadav](mailto:syadav@uchc.edu)_sumit17@yahoo.com

*Corresponding Author: Sumit Yadav, 263 Farmington Avenue, L7063 MC1725, University of Connecticut Health Center, Farmington, CT 06030

**Supplemental Data - Histological Staining**

***TRAP staining***

Sagittal sections of condyles of experimental and control mice were first incubated with the TRAP reaction buffer, which contained sodium acetate anhydrous, sodium L-tartrate dibasic dihydrate and sodium nitrite (all chemicals from Sigma-Aldrich, St. Louis, MO, USA) for 10 min. Slides were then layered with the TRAP substrate, containing the reaction buffer and ELF97 (Life Technologies, Grand Island, NY, USA) for 5 min under UV light. The ELF97 substrate generates a yellow fluorescent signal for TRAP activity. Slides were then coverslipped with 30% glycerol/PBS.

***Edu staining***

We studied cell proliferation by EdU assay. Animals received injections of EdU (5-ethnyl-2’-deoxyuridine, Life Technologies, Grand Island, NY, USA), in a concentration of 30mg/kg body weight, 48 and 24 hours before euthanasia. Subsequently, histological sections were stained using the ClickiT® EdU staining kit according to the manufacturer instructions (ClickiT® EdU Alexa Fluor 555 HCS kit, Life Technologies, Grand Island, NY, USA). Finally, slides were coverslipped with 30% glycerol/PBS and 1/1000 DAPI (Thermo Fisher Scientific, Waltham, MA, USA) for nuclear staining.

***Alkaline phosphatase***

Sections were stained for Alkaline Phosphatase (AP) activity using a fluorescent fast red substrate. Briefly, slides were incubated with the AP buffer, containing Tris (Thermo Fisher Scientific, Waltham, MA USA), MgCl_2_ and NaCl (Sigma-Aldrich, St. Louis, MO, USA) for 10 min. Next, slides were treated with the AP substrate, containing the AP buffer plus Naphtol and Fast Red (Sigma-Aldrich, St. Louis, MO, USA) for 5 min. Slides were then coverslipped with 30% glycerol/PBS and 1/1000 DAPI (Thermo Fisher Scientific, Waltham, MA, USA).

***Toluidine Blue***

Toluidine Blue staining was performed by briefly (14-17 seconds) incubating slides with a solution prepared with Toluidine Blue O (Sigma-Aldrich, St. Louis, MO, USA) and 70% ethanol, at a PH of 2-2.5. Slides were finally coverslipped with 30% glycerol/distilled water.

***Immunostaining for pSMAD158 and VEGF***

Slides were first incubated with antigen retrieval (Epitope retrieval solution, IHC World, LLC; Ellicott City, MD, USA) for 15 min, followed by 0.3% hydrogen peroxide for 30 min. Next, we blocked histological sections for 10 min (Power block ^TM^, Biogenex, Fremont, CA, USA). Slides were then incubated with primary antibody at 4° C overnight. We used a concentration of 2 µm/ml of pSMAD158 antibody (EMD Millipore, Billerica, MA, USA) and also of 2 µm/ml of VEGF antibody (ABCAM, Cambridge, MA, USA). Next day, sections were treated with secondary antibody (VECTASTAIN® Elite ABC system, Vector Laboratories, Burlingame, CA, USA) for 45 min at room temperature. Finally, the immunostaining reaction was precipitated by DAB (3, 3 –diaminobenzidine HRP substrate kit, Vector Laboratories, Burlingame, CA, USA) and counterstained with hematoxylin. Slides were coverslipped with 30% glycerol/distilled water.
